# Healthcare workers’ socio-psychological status after a year with COVID-19 pandemic: a cross-sectional face-to-face survey in Erzincan, Turkey

**DOI:** 10.11604/pamj.2022.41.345.33421

**Published:** 2022-04-28

**Authors:** Hakan Gökalp Taş, Nursel Kuyrukluyildiz, Mustafa Akkus, Ufuk Kuyrukluyildiz

**Affiliations:** 1 Department of Anesthesiology and Reanimation, Siran State Hospital, Gumushane, Turkey,; 2 Vocational School of Health Services, Erzincan Binali Yildirim University, Erzincan, Turkey,; 3 Department of Mental Health and Diseases, Faculty of Medicine, Erzincan Binali Yildirim University, Erzincan, Turkey,; 4 Department of Anesthesiology and Reanimation, Faculty of Medicine, Erzincan Binali Yildirim University, Erzincan, Turkey

**Keywords:** Depression, anxiety, healthcare workers, psycho-social status, COVID-19 pandemic

## Abstract

**Introduction:**

on January 7^th^ 2020, SARS-CoV-2 was identified in Wuhan, China, and on March 11^th^, 2020, the World Health Organization declared it a “Pandemic”. The aim of this research is to assess depression, anxiety, work, and social status in healthcare workers during the COVID-19 pandemic.

**Methods:**

the research was designed to be a cross-sectional face-to-face survey. The study included 111 healthcare employees and 222 non-healthcare workers between the ages of 18 and 65 who applied to the hospital. For some reason, no one was excluded from the research. Socio-demographic and lifestyle-related questions, depression, anxiety, work-social adjustment scores, and pandemic-social status-operation connections were all assessed using a self-report questionnaire containing psychometric measures.

**Results:**

the mean age of the participants in the study was 33.67±10.01 and 59% of the participants were female. PHQ9: 11.67±6.41, GAD7: 9.06±5.81, and W&SAS: 17.55±10.98 were the scores of the healthcare professional groups. PHQ9: 10.25±6.21, GAD7: 7.59±5.65, and W&SAS: 14.75±10.27 were the non-healthcare professional groups' results. When the PHQ9, GAD7, and W&SAS scores of both groups were compared, there was no statistically significant difference in the PHQ9 depression score between the two groups (p=0.107), the GAD7 (p<0.05) and W&SAS (p<0.05) scores of the healthcare professionals were statistically significantly higher.

**Conclusion:**

in comparison to the non-healthcare worker group, healthcare professionals had the same level of depression, greater levels of moderate and high anxiety, and higher levels of work-social adjustment disorder. Unlike the literature, we found that the degree of depression fell to the same level as the non-health professional group in our study, but it was still disadvantaged in terms of anxiety and work-social adjustment.

## Introduction

The Chinese Center for Disease Control and Prevention discovered SARS-CoV-2 in a patient with atypical pneumonia in Wuhan, China, on January 7^th^ 2020, via a nasopharyngeal swab [1]. The World Health Organization declared a “Pandemic” on March 11^th^ 2020 [2]. SARS-CoV-2 quickly spread across the world, with 134,957,021 confirmed cases and 2,918,752 deaths recorded by April 2021 [3]. Because COVID-19 is a highly contagious disease, healthcare workers are at a high risk of infection. One of the early studies in Wuhan found that 29% of patients were healthcare workers [4]. Between February 12^th^ and April 9^th^ 2020, a total of 9,282 healthcare workers were diagnosed with COVID-19, with 27 deaths, according to a report from the American Centers for Disease Control and Prevention (CDC). It has been shown that healthcare workers account for 11-19% of COVID-19 cases [5].

The sudden start of a life-threatening pandemic may put an enormous amount of strain on healthcare professionals, as we have seen in studies of SARS or Ebola epidemics [6]. Increased workload, insufficient personal equipment, physical stress, nosocomial transmission and the need to make ethically challenging decisions can all have a negative impact on healthcare professionals' physical and mental health. Working conditions, along with the threat of disease in their social surroundings and families, can induce mental health issues in healthcare professionals, such as fear and anxiety [7-14].

During the pandemic, depression levels have been found to rise in both healthcare and non-healthcare workers [15]. There are additional research that demonstrate that healthcare personnel have a greater rate of depression [16, 17]. Similarly, anxiety levels increased during the pandemic, and it has been demonstrated in the literature to be greater among healthcare workers in certain studies, and higher in non-healthcare professionals in others [18]. During the pandemic, healthcare professionals' work-social adjustment score was also found to be relatively low [19].

To obtain herd immunity after implementing an efficient vaccination program, the vaccination rate should be as high as possible [9, 10]. One of the obstacles is vaccine hesitancy, which is described as a delay in accepting or refusing immunization despite the availability of vaccination services. It's a complicated particular situation that changes depending on the period, location, and vaccinations used. Factors like peace of mind, convenience, and trust play a role [11, 12]. The purpose of this study was to compare the depression, anxiety, work, and social lives of healthcare professionals to the non-healthcare professionals during the COVID-19 pandemic.

## Methods

**Study design and setting:** the research was designed to be a cross-sectional face-to-face survey. The study began after Erzincan Binali Yildirim University - Clinical Research Ethics Committee approved it (Date: 28.05.2021, Number: 07/13) and the patients gave their written consent. The study population was determined to be Erzincan Mengucek Gazi Training and Research Hospital.

**Study population:** the research comprised 111 healthcare professionals aged 18-65 who were currently working at the hospital and 222 non-health employees aged 18-65 who applied to the hospital. Power analysis was not possible since the study was conducted in a confined universe. The study had to include twice as many non-health professionals in order to be statistically significant. For some reason, no one was excluded from the study.

**Data collection:** data were collected using a self-report questionnaire that included signed informed consent, socio-demographic and lifestyle questions, depression (PHQ9), anxiety (GAD7), work-social adjustment measures (W&SAS), and psychometric scales to examine pandemic-social status-surgery connections.

## Definitions

**Socio-demographic data:** age, gender, monthly income (under 5000 TL/5000-10000 TL/over 10000 TL), education status (none/primary education/ high school/university/higher education), marital status (married/single), people living together (with family/lives alone), friendship relations (good/average/bad), number of children at home, number of children attending school, spouse's employment status (yes/no), presence of caregiver (yes/no), individuals over 65 at home (yes/no), presence of chronic disease (yes/no), presence of individuals with chronic diseases at home (yes/no) were asked to the participants in both groups.

**Patient health questionnaire - PHQ-9:** the individuals in both groups were assessed using a 9-item PHQ9 scale to assess their depression symptoms. This measure has nine items on a four-point scale (Likert scale) ranging from 0 (“Never”) to 3 (“Almost every day”). The severity of depression was divided into five categories: 0-4 (minimum), 5-9 (mild), 10-14 (moderate), 15-19 (moderately severe), and 20-27 (severe).

**Generalized anxiety disorder - GAD-7:** the individuals in both groups were assessed using a 7-item GAD7 scale to assess their anxiety symptoms. This measure is made up of seven items on a four-point scale (Likert scale) ranging from 0 (“Never”) to 3 (“Almost every day”). Anxiety levels were divided into four categories: 0-4 (minimal), 5-9 (mild), 10-14 (moderate), and 15-21 (severe).

**Work and social adjustment scale - W&SAS:** the participants in both groups were given the W&SAS 5-question scale. This measure consists of five items on an eight-point Likert scale, ranging from 0 (“Never”) to 8 (“Always”). A W&SAS score of 20 or more indicated moderately severe or worse psychopathology, a score of 10 to 20 indicated significant functional impairment but less severe clinical symptomatology, and a score of less than 10 indicated a subclinical population.

**Pandemic - social status - surgery relations:** would you like to have surgery with your doctor's recommendation during the pandemic, except for emergencies? (Yes/No), If you decide to have an operation, would you like to have an operation in the pandemic hospital? (Yes/No); have you had a complaint that you hesitated to apply to the hospital for fear of an operation decision during the pandemic process? (Yes/No); do you think the virus that causes COVID-19 infection is a virus that emerged naturally without human intervention? (Yes/No); have you been vaccinated against COVID-19? (Yes/No), COVID - 19 (SARS-COV-2) Have you had an infection? (Yes/No); did you have the infection before you got the COVID-19 Vaccine? (Yes/No); did you complete your inpatient treatment? (Yes/No), If you have not been vaccinated against COVID-19, what is the reason? (6 independent yes/no choices) questions were asked to the participants in both groups.

**Statistical analysis:** the data of 111 hospital employees who took part in the survey, as well as 222 non-hospital employees, were statistically analysed. Results are presented as mean ± standard deviation for continuous variables and numbers for categorical variables. Because the universe under study was the only one, no sample size power analysis was done. IBM SPSS Statistics 22.0 (IBM Software, New York, United States) and Microsoft Office - Excel 2016 programs were used for statistical analysis and calculations.

**Ethical considerations:** before the questionnaire was administered, all patients were told about the study protocol and procedures to be followed, and their signed consent was acquired. Erzincan Binali Yildirim University - Clinical Research Ethics Committee approved the study (Date: 28.05.2021, Number: 07/13) and the patients gave their written consent.

## Results

**General characteristics of the study population:** our study comprised a total of 333 individuals, including 111 health care employees and 222 non-health workers. The mean age of the participants in the study was 33.67±10.01 and 59% of the participants were female. For whatever reason, no one was eliminated from the research. The socio-demographic features of the population under investigation are summarized in Table 1.

**Table 1:** socio-demographic features of the population under study

		n	%
Gender	Male	134	40.2
	Female	199	59.8
Monthly income	<5000	130	39.0
	5000-10000	132	39.6
	>10000	71	21.3
Education status	None	4	1.2
	Primery Education	15	4.5
	High School	25	7.5
	University	213	64.0
	Higher Education	76	22.8
Marital status	Single	144	43.2
	Married	189	56.8
People living together	Living with family	276	82.9
	Living alone	57	17.1
Friendship relations	Good	248	74.5
	Average	79	23.7
	Bad	6	1.8
Number of children at home	0	126	37.8
	1	66	19.8
	2	88	26.4
	>=3	53	15.9
Number of children attending school	0	164	49.2
	1	61	18.3
	2	73	21.9
	>=3	35	10.5
Spouse's employment status	No	114	44.9
	Yes	140	55.1
Presence of caregiver	No	286	85.9
	Yes	47	14.1
Individuals over 65 at home	No	291	87.4
	Yes	42	12.6
Presence of chronic disease	No	274	82.3
	Yes	59	17.7
Presence of individuals with chronic diseases at home	No	241	72.4
	Yes	92	27.6

When all the individuals were looked at, it was discovered that while gender had no influence on GAD7 or W&SAS scores, female gender had a statistically significant higher PHQ9 depression score (p<0.05). While education has no influence on GAD7 or W&SAS scores, the PHQ9 depression score increases statistically significant (p<0.05) as education level increases. The PHQ9, GAD7, and W&SAS scores of those who said they had good friendships were statistically significantly lower than those who said they had moderate or bad connections (p<0.05). While there was no statistically significant rise in PHQ9 and GAD7 scores as the number of children rose, there was a statistically significant increase in W&SAS scores (p<0.05). The GAD7 score had no significant influence as the number of children attending school rose, while the PHQ9 and W&SAS scores increased statistically (p<0.05). The presence of a career raised the PHQ9, GAD7, and W&SAS scores substantially (p<0.05). While having a chronic disease had no effect on the PHQ9 or GAD7 scores, the W&SAS score was statistically significantly higher (p<0.05). The W&SAS score did not change substantially when someone with a chronic disease lived with them, although the PHQ9 and GAD7 levels did (p<0.05). Monthly income, marital status, individuals living together, spouse working status, and an individual over 65 years old living at home had no significant influence on PHQ9, GAD7, and W&SAS scores. The statistical findings of the PHQ9 depression, GAD7 anxiety, and W&SAS work and social adjustment measures in the groups with and without health workers are presented in Table 2.

**Table 2:** PHQ9, GAD7 and W&SAS work and social adjustment scale data

		Healthcare worker			
		No		Yes	
		n	%	n	%
PHQ9 Depression Score*	Minimal	52	23.4	15	13.5
	Mild	54	24.3	35	31.5
	Moderate	55	24.8	23	20.7
	Moderately-Severe	44	19.8	25	22.5
	Severe	17	7.7	13	11.7
GAD7 Anxiety Score**	Minimal	83	37.4	32	28.8
	Mild	60	27.0	29	26.1
	Moderate	50	22.5	28	25.2
	Severe	29	13.1	22	19.8
W&SAS Work and Social Adjustment Score***	Subclinic Population	74	33.3	31	27.9
	Significant functional impairment but less severe clinical symptomatology	84	37.8	30	27.0
	Moderately severe or worse psychopathology	64	28.8	50	45.0

PHQ9: Patient Health Questionnaire 9, GAD7: Generalized Anxiety Disorder 7, W&SAS: Work and Social Adjustment Score. *p=0.053, **p<0.05, ***p<0.05

**Figure 1:**
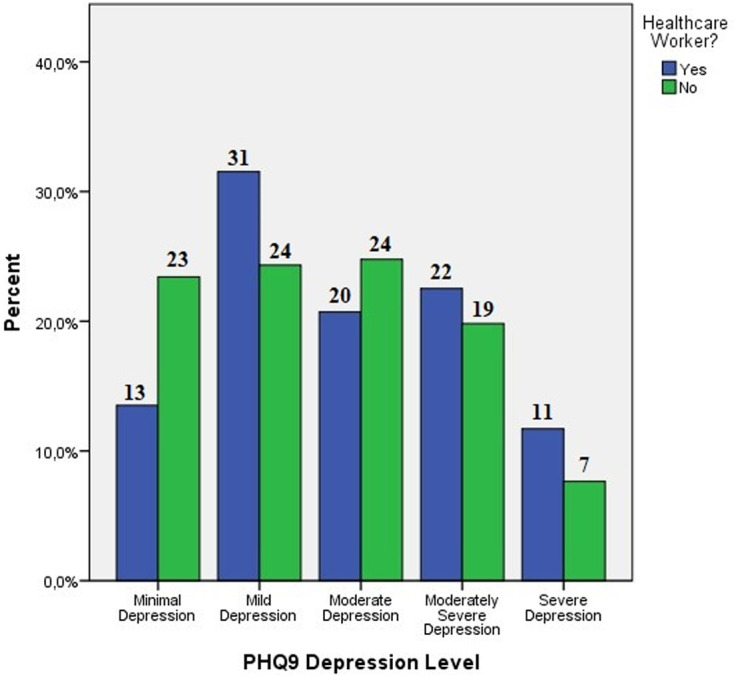
PHQ9 depression level in healthcare professionals and the normal population

**PHQ9 score evaluation:** when the PHQ9 score is evaluated, it was 11.67 ± 6.41 in healthcare workers, 10.25 ± 6.21 in the non-healthcare worker group. It is observed that both the healthcare professional group (54.9%) and the non-healthcare professional group (52.3%) had very high levels of moderate and higher depression. Between the groups with and without healthcare professionals, there was no statistically significant difference in the PHQ9 depression score (p=0.107). Minimal and moderate depression were reported to be higher in the non-healthcare worker group, whereas mild, moderate-severe, and severe depression were found to be more frequent in the healthcare worker group (p<0.05) (Figure 1).

**Figure 2:**
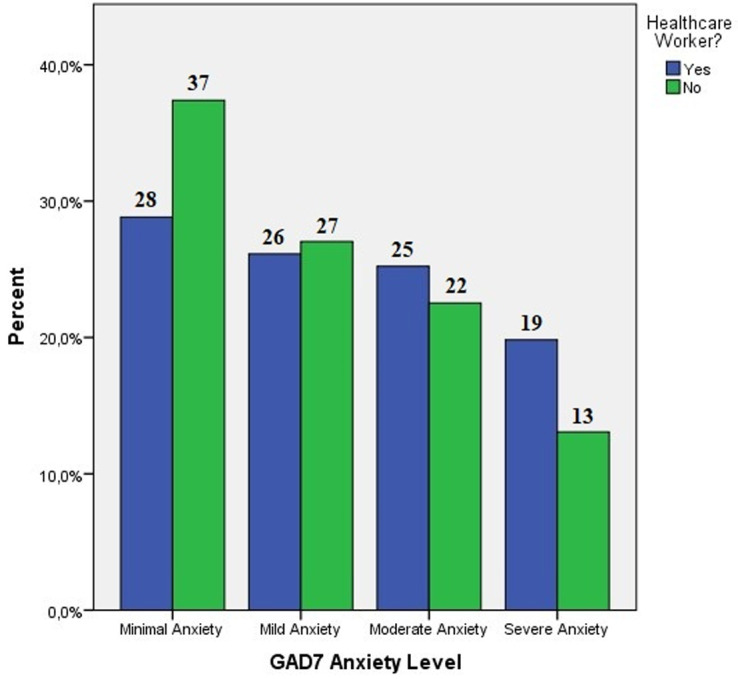
GAD7 anxiety levels in healthcare professionals and the normal population

**GAD7 score evaluation:** when examining at the GAD7 score, it was 9.06 ± 5.81 in healthcare workers, 7.59 ± 5.65 in the non-healthcare worker group. It's apparent that, while healthcare workers have a greater proportion of moderate and higher anxiety (45%), it is indeed at a very high level in both the healthcare worker group and the non-healthcare worker group (35.6%). While the non-healthcare worker group had more minimum and mild anxiety, the healthcare worker group had more moderate and severe anxiety, but the difference was not statistically significant (p=0.258) (Figure 2). The GAD7 anxiety score of healthcare workers was shown to be statistically substantially higher than the normal population (p<0.05) according to the findings of non-parametric analysis.

**Figure 3:**
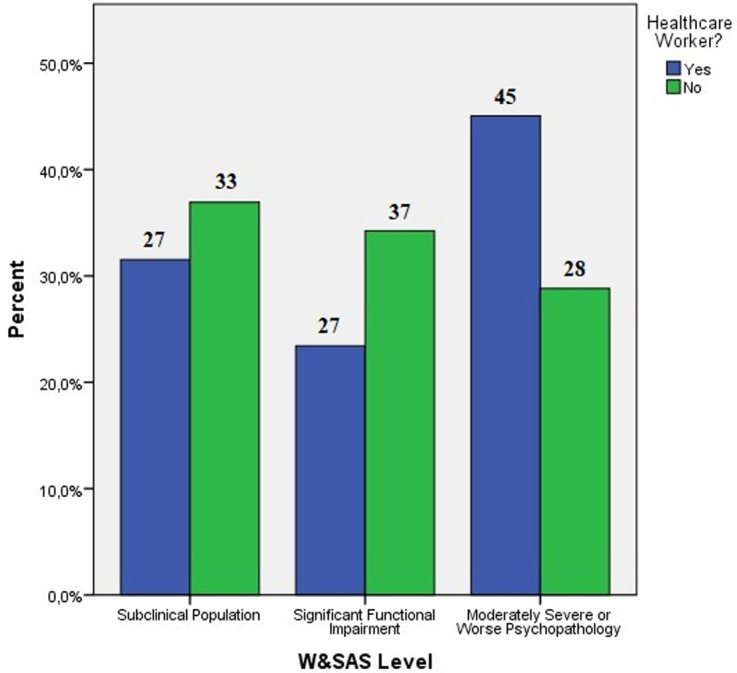
W&SAS levels in healthcare professionals and the normal population

**W&SAS score evaluation:** when looking at the W&SAS score, it was 17.55 ± 10.98 in healthcare workers, 14.75 ± 10.27 in the non-healthcare worker group. It is clear that both the healthcare worker group (72%) and the non-healthcare group have a very high degree of moderate and higher level impairment, which produces functional impairment (66.6%). While the non-healthcare worker group had a statistically significant higher prevalence of subclinical maladjustment and functional impairment with a less severe clinical course, the healthcare worker group had a statistically significant higher risk of severe or worse psychopathology (p<0.05) (Figure 3). When the PHQ9, GAD7, and W&SAS scores of both groups were compared, it was discovered that while there was no statistically significant difference in the PHQ9 depression score between the two groups (p=0.107), the GAD7 (p<0.05) and W&SAS (p<0.05) scores in the healthcare worker group were statistically significantly higher Table 3 summarizes the PHQ9, GAD7, and W&SAS scores.

**Table 3:** PHQ9, GAD7 and W&SAS scores

Healthcare Worker?		PHQ9 Score*	GAD7 Score**	W&SAS Score***
No	Mean	10.25	7.59	14.75
	Std. Deviation	6.215	5.659	10.271
	Median	10.00	7.00	13.50
	Minimum	0	0	0
	Maximum	27	21	40
Yes	Mean	11.67	9.06	17.55
	Std. Deviation	6.418	5.810	10.984
	Median	10.00	9.00	18.00
	Minimum	2	0	0
	Maximum	27	21	40

PHQ9: Patient Health Questionnaire 9, GAD7: Generalized Anxiety Disorder 7, W&SAS: Work and Social Adjustment Score. *p=0.053, **p<0.05, ***p<0.05

## Discussion

One year following the commencement of the COVID-19 pandemic in our nation, we assessed the depression, anxiety, and work-social adjustment of health-care and non-health-care workers in our research. The individuals' depression and anxiety levels were found to be rather high, and their work and social adjustment scores were quite challenging. There was no statistically significant difference between health professionals and non-health care employees when it came to depression. Healthcare employees' anxiety levels were shown to be higher than those of non-healthcare workers (particularly moderate and advanced anxiety). When compared to non-health care professionals, health workers' work and social adjustment were shown to be much worse.

The public's mental health has suffered as a result of the COVID-19 pandemic. When looking through the literature, research suggests that depression and anxiety levels in those impacted by the pandemic might reach 70% [13]. The pandemic's quarantines have also been found to boost depression and anxiety [14]. It has been shown that during pandemics, the likelihood of depression among healthcare professionals rises. Depression ratings of both healthcare professionals and the non-healthcare workers rose during the pandemic period compared to the pre-pandemic period, according to a review of 43 research [15]. Some studies found no difference between groups with and without health professionals in a meta-analysis, while others indicated that health workers had a significantly higher risk of depression [16]. Some studies found no difference between groups with and without health professionals in a meta-analysis, while others indicated that health workers had a significantly higher risk of depression.

Anxiety prevalence in the non-healthcare workers during the pandemic period was reported to range from 6.33 percent to 50.9 percent in a review that included some research [16]. Another systematic review and meta-analysis found that 23.2 percent of healthcare professionals had anxiety during the pandemic timeframe [17]. While some studies suggest that anxiety levels are greater in healthcare professionals during the pandemic, others show that anxiety levels are higher in the non-healthcare workers [18]. While minimal and mild anxiety was proportionally higher in the non-healthcare worker group, moderate and severe anxiety was proportionally higher in the healthcare worker group, but this difference was not statistically significant. This circumstance leads us to believe that anxiousness is a more serious issue for healthcare providers.

When the W&SAS score was evaluated in a study conducted in healthcare professionals during the COVID-19 pandemic in Italy, a substantial decrease in global functionality was shown [19]. It has been demonstrated that during the pandemic phase, there are severe impairments in functioning in the general population [20]. However, no correlation was identified in the literature between the degrees of functional impairment in health professionals and non-health employees throughout the pandemic period. In our study, non-healthcare workers had a greater prevalence of subclinical incompatibility and functional impairment with moderate clinical symptoms, whereas healthcare workers had a higher risk of severe or worse psychopathology (p<0.05). This scenario might be linked to the increased strain placed on healthcare workers as a result of the pandemic.

Female gender was typically regarded as a risk factor connected with depression and anxiety in studies related to the influence of COVID-19 [21]. While there was no gender influence on anxiety scores in our study, there was a statistically significant rise in female gender on depression ratings (p<0.05). We found no significant influence of gender on the W&SAS score in our study, despite the fact that there is no literature on the role of gender in the effect of the pandemic on work and social adjustment. In a review, it was claimed that as education levels grew, sadness and anxiety levels increased, and that low educational status was related with poor mental health in some research [22]. The PHQ9 depression score rose statistically as education level increased (p<0.05) in our study, however, there was no significant influence of education level on GAD7 anxiety level.

The PHQ9 and GAD7 scores of those who said they had good friendship relationships were statistically significantly lower than those who said they had moderate or bad friendship relationships (p<0.05). Solid social support is a result of good relationships. Social support is also linked to lower levels of anxiety and depression [23]. It has been suggested that having a child during the pandemic is linked to reduced levels of depression and anxiety [24]. PHQ9 and GAD7 scores rose statistically substantially (p<0.05) when the number of children increased in our study. The large number of children during the pandemic period can be considered to raise anxiety and have a negative impact on mental health. However, it had no significant influence on the W&SAS score, and work and social adjustment were not significantly altered, according to our research.

There is a link between caregiver strain and anxiety and depression, according to studies [25]. The presence of someone whose care was provided during the pandemic era increased the PHQ9, GAD7, and W&SAS scores substantially (p<0.05) in our research. Concerns regarding the caregiver's health are expected to rise as caring becomes more challenging during the pandemic. Although it has been suggested that having a chronic disease is a risk factor for anxiety and depression [26], research have shown that it is linked to health anxiety but not to depression or anxiety [21]. The presence of chronic disease had no effect on the PHQ9 and GAD7 scores in our research. It was found that the W&SAS score was statistically substantially higher (p<0.05). This demonstrates how the existence of a chronic disease in the pandemic has a detrimental impact on work and social harmony.

Although it has been demonstrated that having a high monthly income and being single are among the characteristics that exacerbate the negative psychological effects of the pandemic [27], our research found no significant influence of monthly income or marital status on PHQ9, GAD7, or W&SAS scores. Despite the fact that our study was designed and performed as a prospective survey, disparities in vaccination rates between healthcare professionals and non-healthcare employees might lead to a shift in public perception of the pandemic. As long as the pandemic circumstances persist, we believe it is critical to examine the present socio-psychological situation by repeating our survey on a regular basis.

## Conclusion

It was discovered that the overall population's depression and anxiety levels were fairly high, and that work and social adjustment were quite challenging. In terms of depression, it was shown that there was no statistically significant difference between health professionals and the non-healthcare workers. Anxiety levels among healthcare professionals were shown to be greater than in the non-healthcare workers (particularly moderate and advanced anxiety). Work and social adjustment were shown to be significantly decreased among health workers when compared to the non-healthcare workers.

### What is known about this topic

- In both society and healthcare professionals, the COVID-19 pandemic has resulted in a surge in psychological disorders like as depression and anxiety;
- The pandemic's deleterious impacts have had a detrimental impact on work and social situations.

### What this study adds

- After a year with the pandemic, it was discovered that the degree of depression had fallen to the same level as the non-healthcare workers, but remained high;
- The degree of anxiety among healthcare professionals was found to be greater than that of the non-healthcare workers;
- According to the W&SAS score, healthcare workers had more severe or worse psychopathology in our study.

## References

[1] Chen N, Zhou M, Dong X, Qu J, Gong F, Han Y (2020). Epidemiological and clinical characteristics of 99 cases of 2019 novel coronavirus pneumonia in Wuhan, China: a descriptive study. Lancet.

[2] Huang C, Wang Y, Li X, Ren L, Zhao J, Hu Y (2020). Clinical features of patients infected with 2019 novel coronavirus in Wuhan, China. Lancet.

[3] World Health Organization (WHO) WHO Coronavirus (COVID-19) Dashboard.

[4] Wang D, Hu B, Hu C, Zhu F, Liu X, Zhang J (2020). Clinical characteristics of 138 hospitalized patients with 2019 novel coronavirus-infected pneumonia in Wuhan, China. JAMA.

[5] CDC COVID-19 Response Team (2020). Characteristics of health care personnel with COVID-19” United States, February 12-April 9, 2020. MMWR Morb Mortal Wkly Rep.

[6] Liu X, Kakade M, Fuller CJ, Fan B, Fang Y, Kong J (2012). Depression after exposure to stressful events: lessons learned from the severe acute respiratory syndrome epidemic. Compr Psychiatry.

[7] Lung FW, Lu YC, Chang YY, Shu BC (2009). Mental symptoms in different health professionals during the SARS attack: a follow-up study. Psychiatr Q.

[8] Wu P, Fang Y, Guan Z, Fan B, Kong J, Yao Z (2009). The psychological impact of the SARS epidemic on hospital employees in China: exposure, risk perception, and altruistic acceptance of risk. Can J Psychiatry.

[9] Fontanet A, Cauchemez SJNRI (2020). COVID-19 herd immunity: where are we?. Nat Rev Immunol.

[10] Ashby B, Best A (2021). Herd immunity. Curr Biol.

[11] Swaminathan S (2020). The WHO´s chief scientist on a year of loss and learning. Nature.

[12] MacDonald NEJV (2015). Vaccine hesitancy: Definition, scope and determinants. Vaccine.

[13] Bouattour W, Turki M, Ellouze S, Messedi N, Charfeddine F, Halouani N (2021). Psychological responses of Tunisian general population during COVID-19 pandemic. Pan Afr Med J.

[14] Kharroubi G, Cherif I, Amor SH, Zribi M, Atigue WB, Ouali U (2021). Mental health status of adults under institutional quarantine: a cross-sectional survey in Tunisia. Pan Afr Med J.

[15] Vindegaard N, Benros ME (2020). COVID-19 pandemic and mental health consequences: Systematic review of the current evidence. Brain Behav Immun.

[16] Wu T, Jia X, Shi H, Niu J, Yin X, Xie J (2021). Prevalence of mental health problems during the COVID-19 pandemic: A systematic review and meta-analysis. J Affect Disord.

[17] Pappa S, Ntella V, Giannakas T, Giannakoulis VG, Papoutsi E, Katsaounou P (2020). Prevalence of depression, anxiety, and insomnia among healthcare workers during the COVID-19 pandemic: A systematic review and meta-analysis. Brain Behav Immun.

[18] Singh RK, Bajpai R, Kaswan P (2021). COVID-19 pandemic and psychological wellbeing among health care workers and general population: A systematic-review and meta-analysis of the current evidence from India. Clin Epidemiol Glob Health.

[19] Carmassi C, Malacarne P, Dell'oste V, Bertelloni CA, Cordone A, Foghi C (2021). Post-traumatic stress disorder, burnout and their impact on global functioning in Italian emergency healthcare workers. Minerva Anestesiol.

[20] Andersen LH, Fallesen P, Bruckner TA (2021). Risk of stress/depression and functional impairment in Denmark immediately following a COVID-19 shutdown. BMC Public Health.

[21] Özdin S, BayrakÖzdin Ş (2020). Levels and predictors of anxiety, depression and health anxiety during COVID-19 pandemic in Turkish society: The importance of gender. Int J Soc Psychiatry.

[22] Hossain MM, Tasnim S, Sultana A, Faizah F, Mazumder H, Zou L (2020). Epidemiology of mental health problems in COVID-19: a review. F1000Res.

[23] Steinsdottir FK, Halldorsdottir H, Gudmundsdottir A, Arnardottir S, Smari J, Arnarson EO (2008). [Diabetes type 1 in young adults: The relationship between psycho-social variables, glycemic control, depression and anxiety]. Laeknabladid.

[24] Elbay RY, Kurtulmuş A, Arpacıoğlu S, Karadere E (2020). Depression, anxiety, stress levels of physicians and associated factors in COVID-19 pandemics. Psychiatry Res.

[25] Hu P, Yang Q, Kong L, Hu L, Zeng L (2018). Relationship between the anxiety/depression and care burden of the major caregiver of stroke patients. Medicine (Baltimore).

[26] Picaza Gorrochategi M, Eiguren Munitis A, Dosil Santamaria M, Ozamiz Etxebarria N (2020). Stress, Anxiety, and Depression in People Aged Over 60 in the COVID-19 Outbreak in a Sample Collected in Northern Spain. Am J Geriatr Psychiatry.

[27] Cansel N, Ucuz İ, Arslan AK, KayhanTetik B, Colak C, Melez Ş (2021). Prevalence and predictors of psychological response during immediate COVID-19 pandemic. Int J Clin Pract.

